# Clinical remission of disseminated molluscum contagiosum infection in a patient with atopic dermatitis treated with dupilumab^☆^

**DOI:** 10.1016/j.abd.2021.10.001

**Published:** 2022-03-17

**Authors:** Marta Elosua-González, Ángel Rosell-Díaz, Fernando Alfageme-Roldán, Mercedes Sigüenza-Sanz, Gaston Roustan-Gullón

**Affiliations:** Department of Dermatology, Hospital Universitario Puerta De Hierro, Majadahonda, Madrid, Spain

**Keywords:** Alopecia areata, Dermatitis, atopic, Molluscum contagiosum, Therapy, Treatment

## Abstract

Atopic dermatitis predisposes to skin infections, and on the other hand, some therapies used for atopic dermatitis may worsen viral infections whose lesions may be more diffuse and resistant to treatment. The authors present a patient with severe atopic dermatitis and disseminated molluscum contagiosum infection. The molluscum contagiosum did not clear with topical treatment, and it worsened her atopic dermatitis even more, so the authors started treatment with dupilumab. After two months, the patient's dermatitis went into clinical remission and there was resolution of the infection with no recurrence at the 12-month follow-up. Dupilumab is nowadays a promising treatment for severe atopic dermatitis. To our knowledge, only four reports of molluscum contagiosum during dupilumab therapy have been reported in the literature, with contrasting effects. According to the authors’ experience, treatment with dupilumab appears to be a safe alternative for patients with severe atopic dermatitis who are also infected with molluscum contagiosum, as opposed to other treatments such as systemic corticosteroids or cyclosporine.

## Introduction

Atopic dermatitis (AD) predisposes to skin infections such as *Staphylococcus aureu*s, herpes simplex virus, and molluscum contagiosum (MC) infection. MC is often a self-limited viral infection with pathogenesis poorly understood. It is known that some therapies used for AD worsen MC infection that may present with lesions that are diffuse, resistant to treatment, and persistent over time. Here, the authors describe an AD patient with widespread MC lesions who experienced clearance of the infection shortly after treatment of AD with dupilumab.

## Case report

**Figure 1:**
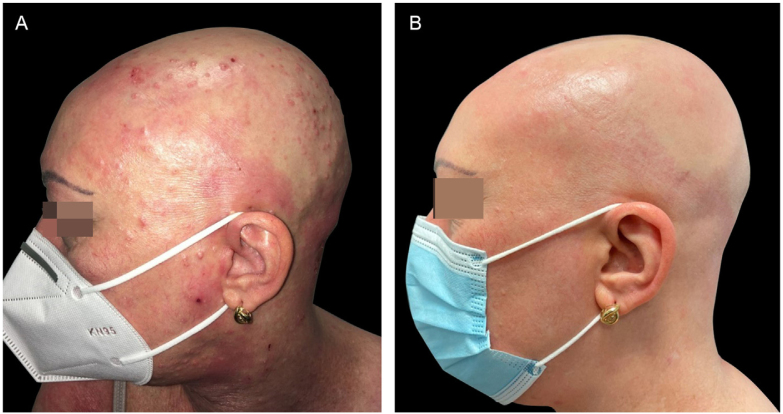
(A), The patient with severe AD, alopecia areata and molluscum contagiosum infection before treatment with dupilumab. Excoriation, eczematous lesions, and dome-shaped papules with central umbilication widespread all over the body but especially on her face and scalp. (B), Two months after starting treatment with dupilumab. Clinical remission of atopic dermatitis and resolution of molluscum contagiosum infection.

**Figure 2:**
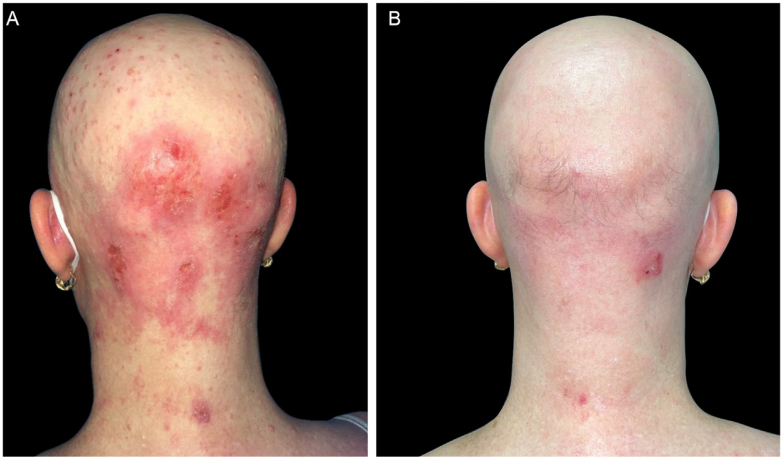
(A), The patient with severe AD, alopecia areata and molluscum contagiosum infection before treatment with dupilumab. Excoriation, eczematous lesions, and dome-shaped papules with central umbilication widespread all over the body but especially on her face and scalp. (B), Two months after starting treatment with dupilumab. Clinical remission of atopic dermatitis and resolution of molluscum contagiosum infection.

**Figure 3:**
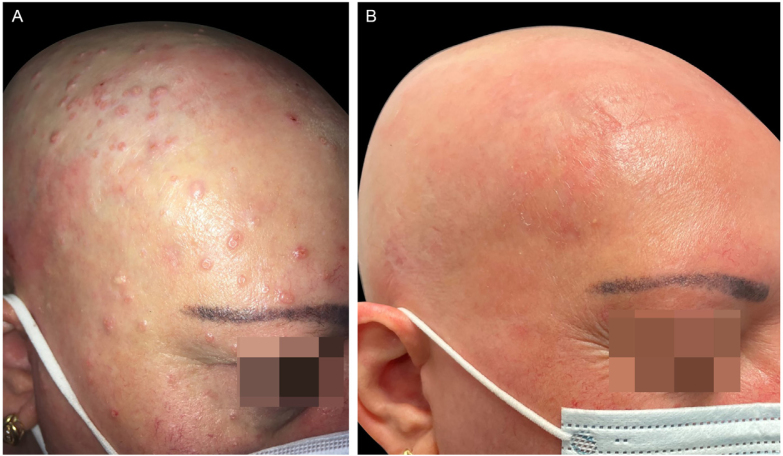
(A), The patient with severe AD, alopecia areata and molluscum contagiosum infection before treatment with dupilumab. Excoriation, eczematous lesions, and dome-shaped papules with central umbilication widespread all over the body but especially on her face and scalp. (B), Two months after starting treatment with dupilumab. Clinical remission of atopic dermatitis and resolution of molluscum contagiosum infection.

A 47-year-old woman came to the present study’s clinic with severe AD since childhood and a 16-year history of alopecia areata to start treatment with dupilumab (a monoclonal antibody that inhibits both interleukin-4 and interleukin-13). She had been treated several years ago with topical and systemic corticosteroids, phototherapy, and cyclosporine with no improvement. During her physical examination, she had severe AD with Eczema Area and Severity Index (EASI) 24.7, Scoring Atopic Dermatitis (SCORAD) 60.26, and Body Surface Area (BSA) 30. Unexpectedly, she also had dome-shaped papules with central umbilication consistent with MC widespread all over the body but especially on her face and scalp, which had appeared 2-months previously (Figure 1, Figure 2, Figure 3). Laboratory studies did not reveal any abnormalities except high levels of IgE. HIV infection and hyper IgE syndrome were ruled out. Topical treatment with 10% potassium hydroxide solution and 5% imiquimod cream was initiated, worsening both the AD (EASI 38.5, SCORAD 76.82, BSA 70) and the MC. Therefore, the authors started treatment for AD with dupilumab and continued with topical treatment for MC associating curettage in the areas with more lesions. After two months, AD went into clinical remission (EASI 1, BSA 1) with a resolution of MC as well (Figs. 1‒3), including the lesions that did not receive any topical treatment or curettage. Alopecia areata did not improve during the treatment. The patient continued with dupilumab treatment with no recurrence after a 12-months follow-up.

## Discussion

AD patients are at an increased risk of disseminated MC, probably because of locally impaired cell-mediated immunity due to a dominant T helper 2 (Th2)-mediated immune response in the skin, impaired epidermal barrier function, alterations in the skin microbiome, autoinoculation associated with scratching and the use of immunosuppressive therapies.^1^, ^2^

To our knowledge, only four reports of disseminated MC during dupilumab therapy have been reported with contrasting effects. Three of them 1, 2, 3 describe a total of six patients with AD and concomitant MC infection, which started treatment with dupilumab. In all of them, complete resolution of MC was achieved during treatment with dupilumab, although some cases experienced an initial inflammation or even dissemination of the MC. However, Sevray et al. ^4^ reported a case of a 47-year-old patient with AD and alopecia areata, which both improved after eight weeks of treatment with dupilumab, but worsened his previous MC infection. Then dupilumab was stopped, and MC regressed partially after six months. Regarding this case, Di Lernia et al.^3^ propose that dupilumab withdrawal may have prevented the observation of subsequent clearance of MC. They also consider that partial regression of MC six months after stopping dupilumab, cannot be regarded as proof of drug causality, as suggested by the authors, due to the nature of the infection itself, with spontaneous resolution within months.

Dupilumab inhibits IL-4 and IL-13, thus inhibiting the Th2 immune response that plays a key role in atopic diseases.^4^ It may affect host responses to helminth infections, but additional infections have not been identified.^3^ On the other hand, Th2 cytokines are able to inhibit anti-infective immunity, and their enhanced expression may be a selective advantage for the virus.^3^ Therefore, the selective inhibition of the Th2 pathway induces a Th1/Th2 balance, a 'normalization' of the skin barrier, and permits effective innate and cell-mediated mechanisms to clear the MC.^1^

In the studied patient, there was already initial dissemination of MC with no response to topical treatment. Due to the worsening of AD, the authors started treatment with dupilumab, obtaining a prompt remission of both AD and the infection without any acute inflammation of MC lesions. In summary, according to the literature and our experience, treatment with dupilumab appears to be a safe alternative in AD with extensive MC infection.

## Financial support

None declared.

## Authors' contributions

Marta Elosua-González: Had full access to all of the data in the study and took responsibility for the integrity of the data and the accuracy of the data analysis; drafting and editing of the manuscript; critical review of the literature; critical review of the manuscript.

Ángel Rosell-Díaz: Collection, analysis, and interpretation of data; critical review of the literature; design and planning of the study.

Fernando Alfageme-Roldán: Collection, analysis, and interpretation of data.

Mercedes Sigüenza-Sanz: Drafting and editing of the manuscript; critical review of the manuscript; approval of the final version of the manuscript.

Gaston Roustan-Gullón: Collection, analysis, and interpretation of data; critical review of the literature.

## Conflicts of interest

None declared.
